# Spontaneous and Induced Tumors in Germ-Free Animals: A General Review

**DOI:** 10.3390/medicina57030260

**Published:** 2021-03-11

**Authors:** Rajbardhan Mishra, Lenka Rajsiglová, Pavol Lukáč, Paolo Tenti, Peter Šima, Fabián Čaja, Luca Vannucci

**Affiliations:** 1 Laboratory of Immunotherapy, Institute of Microbiology v.v.i., Czech Academy of Sciences, Videnska 1083, 14220 Prague, Czech Republic; immuno@biomed.cas.cz (R.M.); rajsiglova@biomed.cas.cz (L.R.); pavol.lukac@biomed.cas.cz (P.L.); paolo.tenti@biomed.cas.cz (P.T.); sima@biomed.cas.cz (P.Š.); caja@biomed.cas.cz (F.Č.); 2 Faculty of Science, Charles University, Albertov 6, 12800 Prague, Czech Republic

**Keywords:** germ-free animals, microbiome, spontaneous tumors, induced tumors, colorectal cancer

## Abstract

Cancer, bacteria, and immunity relationships are much-debated topics in the last decade. Microbiome’s importance for metabolic and immunologic modulation of the organism adaptation and responses has become progressively evident, and models to study these relationships, especially about carcinogenesis, have acquired primary importance. The availability of germ-free (GF) animals, i.e., animals born and maintained under completely sterile conditions avoiding the microbiome development offers a unique tool to investigate the role that bacteria can have in carcinogenesis and tumor development. The comparison between GF animals with the conventional (CV) counterpart with microbiome can help to evidence conditions and mechanisms directly involving bacterial activities in the modulation of carcinogenesis processes. Here, we review the literature about spontaneous cancer and cancer modeling in GF animals since the early studies, trying to offer a practical overview on the argument.

## 1. Introduction

Germ-free (GF) animals are a crucial model to assess the interactions between a host and its microbiota. GF animals are also known as axenic, referring to experimental animals completely free from all kinds of microbes since their birth, and continuously maintained under protected conditions (sterile incubators). The GF animals represent a potent biological model developed to study the role of bacteria on immunity, its responses and evolution [1,2,3,4,5]. They are also usedfor preparing specific germcontaminated animals (gnotobiotic model) to evaluate the capability of one or more associated bacteria to induce and support the development of immune responses and illnesses, e.g., cancer [6,7]. The presence of commensal bacteria in the gut maintains a continuous stimulus sustaining a constant activation of the immunity in the mucosa, and, contemporarily, it has metabolic effects by the elaboration of the bile and food components as well [8,9,10]. Therefore, the absence of such immunological stimulation and metabolic interactions in GF animals makes them a unique tool to study the influence that bacteria can have in the development of colorectal cancer (CRC) [11,12,13]. Thus, since the beginning of GF animal production, many studies have been done using this model to demonstrate that certain types of microbiota can activate inflammatory and oncogenic pathways and promote intestinal tumorigenesis leading to CRC [11,12,14,15,16,17]. Dietary habits can affect the composition of intestinal microbiota leading to dysbiosis and consequent pathological changes in the colon mucosa [13,18,19,20]. As such, dietary habits, and associated microbiota, represent an essential environmental factor implied in CRC development.

The intestinal microflora composition at present looks a critical factor for the induction of cancer and its prevention [17,20,21,22,23]. The development of the concept of microbiome, and the studies about the complete complex of bacteria regularly colonizing our body since birth, has evidenced its broader involvement in human health maintenance. The microbiome can influence multiple aspects of the immunity and metabolism in health and disease [24,25]. The increasing recourse to studies on GF animals is granting access to a better understanding of these aspects, allowing to discriminate which pathologic mechanisms are modulated by the microbiome composition and which not, particularly in gut diseases like the CRC.

In this review, we will discuss the development and localization of different kinds of spontaneous and induced tumors in GF animals. To understand the missing role of microbes in GF animals, tumor incidences were compared between GF and their conventional (CV) counterparts [26]. Special attention was given to CRC incidences in various experimental models.

The spontaneous tumor occurrence was a topic investigated in the early period of GF animal model development (especially between the 1960s and 1980s) followed by development of colorectal carcinogenesis models. Pollard and Reddy were the researchers that more dedicated their activity to these investigations, starting from the observation of the spontaneous onset of tumors in GF and progressively developing models of comparative carcinogenesis in GF versus CV animals.

## 2. Spontaneous Tumours in GF Animals

Occurrence of spontaneous neoplasms was observed in GF animals. In 1963 Pollard and Teah evaluated the onset of spontaneous tumors in GF rats within the groups of control animals used for other experiments but not as a part of specific evaluation over an established survival period. The rat strains were Wistar, Fischer and the Sprague-Dawley propagated until 8, 3, and 2 years, respectively. On the bulk of 359 control rats used for various experiments, they found spontaneous tumors only in the Wistar strain. The prevalence was breast cancer both in males and females, occurring since the age of six months, until 38 months, according to the period of sampling. The highest incidence of tumor was found between 14 and 28 months, with the highest number at the 25th month. Twenty-five spontaneous tumors were observed; 18 involved the mammary gland and 3 the thymus. The thymic tumors were lymphosarcoma, while breast tumors were mostly adenofibroma, and a few were adenocarcinoma. Invasion of these tumors was not seen into any organ [27]. Seven years later, Pollard and Kajima repeated a similar study on GF Wistar rats (>2 years old) with a different result than in the previous research. They observed a lower occurrence of tumors that was explained by the modification of the balanced diet for the GF animals introduced after the first study. No one was malignant [28]. Spontaneous neoplasm development was also known to occur in many CV (with microbiome) rats and mice. Prejean et al. found an incidence of 45% in rats and 26% in mice, in animals used as a control in various experiments extended up to 18 months. Tumors occurred in various organs, with prevalence of endocrine tumors in the rats and lung tumors in the mice [29].

In male GF Wistar rats were also observed spontaneously developed malignant prostate tumors able to reach a large size, even occupying most of the pelvic cavity. Some of the rats had metastatic lesions in many organs such as spleen, liver, kidney, pancreas, and part of the intestine. They were found between 22 to 40 months of age. [30]. A comparison with the period of incidence of mammary tumors observed in the same GF rats, suggests an age-related sensitivity to variation in the hormonal asset similar to humans, apparently microbiome independent.

Spontaneous mammary tumor in GF C3H mice was seen at the average age of 19.4 months. This study compared the incidence of mammary tumors between GF and CV mice and did not find any significant difference [31]. In another study, a colony of Fisher 344 (F344) GF rats was observed for various generations along a period of ten years), and several types of spontaneously developed neoplasms were found in the animals. A total of 180 rats were observed, 78 males and 102 females. Mammary tumors were the second most frequent neoplasms, found in 9 males (11.5%) and 20 females (19.6%), while leukaemia resulted the most predominant malignancy, occurring in 25.6% males and 36% females. The mammary tumors were mainly fibro-sarcoma and adenocarcinoma. Beginning at 500 days, the incidence of mammary tumours and leukaemia in both genders was also increasing with the age. Other types of neoplasms that were found included pulmonary carcinoma, hepatoma, uterine adeno-fibroma, interstitial tumor of the testis, auricular chondrosarcoma and renal tumor [12].

Smith and Pilgrim showed spontaneous tumors in Bagg Albino/c (BALB/c)Pi GF mice. Lung tumors were observed in 21% of mice with the highest incidence in females. Mammary tumor was only in 3% of mice. Moreover, lymphosarcoma (4.5%), reticulum cell sarcoma, skin carcinoma, adrenal cortical carcinoma (3% each), and carcinoma of the ovary (1.5% of female mice) were also observed. An important finding was the association of different type of tumors in the same animals (lung and mammary tumors; lung, ovary and lymphosarcoma).The median age of mice was 21 months [32].

Spontaneous leukaemia developed in GF AK mice at the mean age of 8.7 months. In these leukemic mice, lymphatic organs such as thymus, lymph nodes, and spleen increased up to eight times of their normal size. Others referred the presence of some virus-like particles evident in the cytoplasmic vacuoles of thymoma cells, and in the intercellular spaces of thymus and spleen tissues. These particles were indicated as the possible leukemia agent that might have transmitted to the next generation by congenital route. Notably, there were no differences in the tumor characteristics with CV AK mice [33].

An early sign of colon cancer is intestinal polyposis. This was observed to spontaneously occur in BALB/c GF mice. One-year old mice were sacrificed and polyposis of adenomatous type was found mostly distributed in the small intestine. Moreover, the incidence of polyposis was higher in GF mice as compared to its CV counterpart (GF 68% in female and 89% in male versus CV 37% in female and 51% in male). This suggested that the presence of microflora might suppress the development of polyposis [34]. However, this is not true for other kind of tumors.

The effect of intestinal microflora on the incidence of spontaneous hepatic tumors was studied on C3H/He male mice. At 48 weeks, 39% of GF and 82% of CV mice had hepatic tumors. However, in gnotobiotic mice inoculated with single bacterial species, tumor incidences varied from 62 to 100% according to the chosen bacteria (Escherichia coli, Streptococcus faecalis, Bifidobacterium adolescentis, Bifidobacterium infantis, Clostridium indolis, C. paraputrificum, C. perfringens, C. innocuum, C. nexile, C. ramosum, C. clostridiiforme, Bacteroides multiacidus, Bacteroides fragilis, Veillonella alcalescens, V. parvula, and Lactobacillus acidophilus.). Moreover, hepatic tumor rates varied from 46 to 95% when gnotobiotic mice were inoculated with 2 to 14 bacterial species, indicating that the composition and variety of the colonizing bacteria may behave and influence differently the metabolic functions [35]. Data about CV and GF spontaneous tumors are summarized in Table 1 and Table 2.

## 3. Induced Tumors in Germ-Free Animals

### 3.1. Type and Incidence of Cancer in GF Animals by Chemical Carcinogens

Several organic or inorganic chemicals already used in CV modeling of cancer havebeen used for the induction of tumors in GF animals.

The 7, 12-dimethylbenz(a)-anthracene (DMBA) was administered intraperitoneally together with 15% fat emulsion at 2-weeks interval to 30-days-old male GF and CV rats of Sprague-Dawley and Fischer strains. DMBA is a polycyclic aromatic hydrocarbon with both carcinogenetic and lymphotoxic activities. Two GF Fischer rats that survived 130 days after last DMBA administration developed specific lesions of myeloid leukemia similar to GF Sprague-Dawley and their CV counterparts. However, out of 16 DMBA injected GF Sprague-Dawley rats, 9 had mammary tumors, and 1 had carcinoma of the ear canal [36]. When 20 mg DMBA in sesame oil was fed by stomach tube to GF Sprague Dawley rats, breast tumors developed in 90% of animals, and few had lesions of myeloid leukemia [37].

Four strains of GF mice [Swiss-Webster, (Institute of Cancer Research USA) ICR, C3H and Carworth Farms-Webster) CFW] and two strains of GF rats (Wistar rats and Fischer) inoculated subcutaneously with 0.5 and 1 mg of 3-Methylcholanthrene (3-MCA) in olive oil, respectively. 3-MCA, a polycyclic aromatic hydrocarbon, is a mutagen with carcinogenetic effect due to its covalent binding to DNA. Tumour development was observed in 76% GF mice and 90% GF rats. The tumour were fibrosarcoma in GF mice. One of the main observations was that in GF and CV animals the time after MCA inoculation and appearance of tumors did not differ significantly. The tumour types and its locations in GF animals were similar to those induced by the same agent in conventional control animals. From the result, it is evident that the microbiome does not play a role in chemical carcinogenesis [38].

When 0.1 mL of 0.8% solution of 20-MCA was given orally in olive oil to five to six weeks old GF RFM mice, the occurrence of thymic lymphoma was observed. However, the incidence of myeloid leukemia and skin carcinoma were high when 5% solution of croton oil in benzene was applied on the abdominal skin of mice associated with oral treatment with MCA. Incidence of pulmonary adenomas was seen after both kinds of treatment with MCA [39]. Subcutaneous injection of 0.1 mg of MCA in olive oil induced lung adenomas in GF new-born mice of Swiss-Webster strain. Moreover, a higher number of lung tumors was seen in the GF than in the CV mice [40]. This also pose the question of the age of induction and the effect of immune system maturation activated by the microbiota in the CV animals.

Pulmonary tumor was observed in 56% of GF BALB/c female mice versus 85% in CV mice, when they were given 20 mg of urethane intraperitoneally. Urethane (ethyl carbamate) is considered to develop its carcinogenetic effect by production of epoxides, which damage the DNA. Its effect is documented especially in animals (rodent models). However, none of the GF C3H/HeN, and C57 Black (C57BL)/6N mice had any type of tumor after the same treatment [41]. Genetic susceptibility is another critical factor in relation to the used animal strain and the possible variability of results on tumor incidence reported in the studies.

When mineral oil was given intraperitoneally to GF and CV BALB/c AnN (An line, National Institute of Health, USA) mice, only 6% of GF mice developed plasma cell tumors (70% in CV) and pulmonary adenoma (5% in CV). Moreover, in 48% of GF mice (10% in CV), the onset of pleomorphic reticulum cell sarcoma was seen [42].

From the above data, it is evident that the carcinogenesis induction generated by these chemicals was independent from the presence of microbiome (Table 3).

### 3.2. Role of Carcinogens, Diet and Bile in the Development of CRC in GF Animals

#### 3.2.1. Carcinogens

Methylnitronitrosoguanidine (MNNG) is one of the carcinogens used for several experimental tumor models. It works producing alkylation of guanine and thymine with transition mutations between GC and AT at DNA level. Intra-rectal injection of MNNG for 20 weeks at a dose of 1–3 mg/rat/week into 50 days old female CD Fischer GF and CV rats induced multiple colonic tumors in all rats of both groups. However, colon adenomas were doubled in GF rats compared to CV animals. None of the organs, including the small intestine and cecum, were seen with any tumors [43].When GF and CV male Wistar rats received gavage with 100 µg/mL MNNG, a noticeable difference in tumor incidence was seen. Tumors occurred in only 17% of the GF rats versus 91% of the CV rats. Adenocarcinoma, carcinosarcoma, and adenoma (3.3% each) were developed in three different MNNG treated GF rats. While in GF rats multiple tumors did not develop, they were observed in 8 CV rats (24%). Microscopically, tumors in both groups of animals were glandular type, extended to the submucosa and muscularis, occasionally infiltrating the serosa [44].Bracken fern (Pteridium aquilinum) is known for causing intestinal and urinary bladder neoplasm when fed to rats and cows [45,46,47]. In 1981, a study was performed using GF and CV Wistar female rats to elucidate if the gut microflora plays any role in the induction of tumors by bracken carcinogen. Intestinal tumors were observed in 90% of GF and 94% in CV rats, with localization in the distal portion of the ileum. In GF rats, ileal sarcoma metastasized to the liver. Moreover, none of the GF rats had adenocarcinoma; however, 70% of GF rats had adenomas, and only 50% of CV animals were observed with adenomas. Adrenocortical adenoma was observed in 10% of treated animals. Metastatic lesions were observed only in a GF rats, suggesting that gut microflora does not play a major role in the tumor induction by bracken carcinogen. However, it is noteworthy that if GF and CV rats were both fed with the same amount of bracken diet, a higher rate of intestinal sarcoma was seen in GF rats. The probable reason of this finding is the activity of the gut microflora, which might be involved in the degradation of bracken carcinogen in CV rats [48].

#### 3.2.2. Bile Acids

Dietary effects on the development of CRC have been studied for a long time. They are possibly mediated by changes in intestinal microflora and composition of bile acids and cholesterol metabolites in the colon content [49,50,51,52]. Experiments of animal induction with 1,2 dimethyalhydrazine (DMH) have shown that a fat-rich diet stimulates the secretion of more bile acids and cholesterol metabolites and can promote CRC development in comparison to animals similarly induced but fed with a regular diet [15,53,54].

Sodium deoxycholate (DC) is one of the secondary bil acids metabolic product of intestinal bacteria. When GF Fischer rats were given intrarectal (i.r.) MNNG, and MNNG plus sodium deoxycholate, DC induced multiple colon tumors in about 89% and 82% of rats, respectively. Moreover, when the rats were given MNNG plus DC developed a higher number of adenocarcinomas than the rats given MNNG only [14].

The promoting effect of sodium lithocholate in colon tumor development was evaluated in GF rats, when i.r. administration of MNNG was followed by lithocholate. The results showed that lithocholate acts as colon tumor promoter even without being further modified by the gut microflora. In this case, tumors were localized in the distal part of the large bowel [55].

The direct effect of biliary acids, namely sodium cholate and sodium chenodeoxycholate, was elucidated in CRC development in GF rats. None of the animals were observed with CRC after i.r. administration of these bile acids. A higher but not significant rate of colon tumors was observed in the GF group when animals were administered with a carcinogen MNNG plus cholic acid or MNNG plus chenodeoxycholic acid, as compared with the animals which were given MNNG only. The groups given MNNG only, developed fewer adenocarcinomas and adenomas than rats given MNNG plus cholic acid or chenodeoxycholic. An important finding of this study was that GF animals after MNNG plus cholic acid or chenodeoxycholic acid administration had a lower incidence of colon tumors than the similarly treated CV rats. Since these bile acids alone did not produce colon tumors in either GF or CV animal, it was concluded that these bile acids are not carcinogenic, or are weakly carcinogenic per se [16].

**Figure 1:**
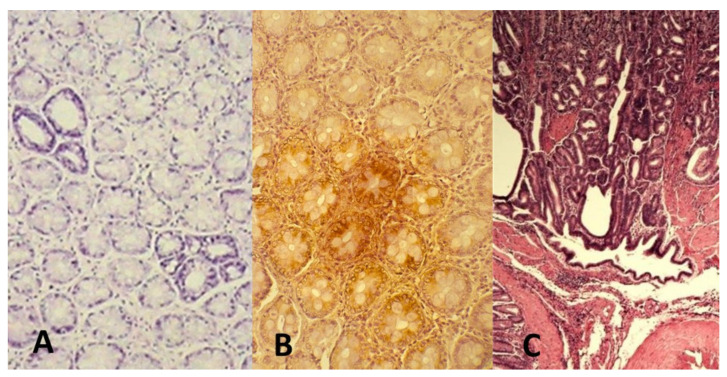
AOM-induced CRC in rat. AOM is an azide compound, active derivative of DMH and induces genetic damage in the colon cells. The carcinogen promotes classical colon carcinogenesis events with adenomas to carcinoma progression [56]. (**A**) aberrant crypts with (**B**) overexpression of p21 k-ras protein (dark brown) and (**C**) development of infiltrating adenocarcinoma after 32 weeks from the induction (original microphotographs from Luca Vannucci’s Lab archive).

In an experimental model by Vannucci et al., where animals were surgically given cecal hernia, azoxymethane (AOM) and porcine bile with high content of secondary biliary salts were used as CRC inducer and promoter, respectively, in GF and CV Wistar-AVN (F 89) rats. AOM (inducer) is an active metabolite of DMH, effective carcinogen on the colon mucosa (Figure 1). The purpose of cecal hernia is to allow direct and exclusive administration and flow of bile acid (promoter) to the complete colon [26,56]. Only 50% of GF and 77.77% of CV animals were observed with cancers. CV animals had multiple cancers as well as tumors of larger size. Mucinous and intestinal-type of adenocarcinomas were present; however, metastasis were not seen in any group at the evaluation after 32 weeks. This study was also focusing on systemic immune response after CRC development. The main difference was observed in the cytotoxic activity of immune cells, higher in GF than in CV animals, especially in rats resistant to the CRC induction. T-lymphocytes in the blood of CRC-bearing animals were 27% higher than in the control GF rats. In this model, the absence of microbiota continuous antigenic challenge and of their metabolic activity collaborated to lower sensitivity to CRC induction as well as to a more active anti-cancer immune response than in CV rats [26].

#### 3.2.3. Microbiome

It has been shown that the intestinal microflora plays a direct role in colon carcinogenesis by 1, 2-dimethylhydrazine (DMH). When CD Fischer rats both GF and CV weresubcutaneously injected with DMH for 20 weeks, none of the GF rats showed colon tumours, whereas 17% of the CV rats treated similarly developed adenocarcinomas of the colon. At 20 weeks after last DMH injection (40 weeks total), 11% of GF rats had developed adenomas, whereas 25% of the CV rats showed colonic tumors. Moreover, induction time for the development of colon tumors by DMH was longer in GF rats compared to CV rats. GF rats were, therefore, less susceptible to develop colon cancer after DMH administration. Indeed, DMH requires activation for producing carcinogenic effects, and supports the concept that the local activation of the carcinogen can be due to the intestinal microflora [54]. DMH, in fact, even in higher doses, had only a limited effect in GF rats, showing its dependence on the microflora for a full activity [11]. Biliary acids represent important factors to assist colonic carcinogenesis and their metabolism is also in relation with the microflora constitution. High incidence of colon cancer is associated with elevated level of fecal bile acid concentrations [50,57,58,59]. Secondary bile acids such as deoxycholic acid and lithocholic acid are the most prevalent bile acids in the etiology of colon cancer in humans [60]. Furthermore patients with colorectal adenomas have a higher deoxycholate level in their serum [61,62]. Lithocholic acid and taurodeoxycholic acid, when given i.r., were found to exert tumor-promoting or accelerating activity in the colon of rats initiated with a sample dose of MNNG [63]. A further indication of the importance of bile acids and their microbiota products, comes from a recent study showing that the metabolism of biliary acids by the intestinal microbiome has effects also on the mucosal immune regulatory cell homeostasis [64].

#### 3.2.4. Enzymes

Another factor to take into consideration is the volume of enzymes present in the colon mucosa, which modulate the capacity of the animal to metabolize the carcinogen DMH [65]. Available evidence indicates that activation of many of the intestinal mucosal enzymes was substantially higher in the GF animals due to the absence of partial inactivation of these enzymes by bacterial products [66,67]. Since these data show that mucosal enzymes are connected to the metabolism of the carcinogen DMH, their activity in GF animals is of interest. In another study, GF and CV rats were given DMH i.r, and azoxymethane (AOM), the activated form of DMH, subcutaneously. In the case of DMH, only 43% of GF animals developed colon tumors in contrast with the 86% of CV. However, the percentage of distribution of adenomas and adenocarcinomas in the colon was similar in both GF and CV rats. In GF rats, small intestinal tumors were found in duodenum at about 2 to 4 cm from the gastroduodenal junction, whereas in CV animals 80% of them occurred in the duodenum, and the remaining 20% occurred in the proximal jejunum. There was no difference in the incidence of AOM-induced neoplasms of the ear duct, kidney, and small intestine between GF and CV rats [68]. This study shows that i.r. administration of DMH induced both small intestinal and large bowel tumors in GF and CV Fischer rats. It seems that DMH or AOM and its metabolites can reach the liver after absorption and may reach the intestine by bile or blood stream [69].

All the above discussed data (Table 4) indicate the complexity of the role that microbiome can plays on the carcinogen efficiency if it needs to be metabolized in the bowel or it is released with the bile after hepatic activation. The elaboration and composition of the bile and the prevalence of specific biliary acids in relation to diet habits, with, consequent effects on the intestinal microbiome composition plays also a not secondary role.

### 3.3. Dextran Sulphate Sodium (DSS) Colitis and Carcinogenesis in GF Animals.

Induction of colitis using dextran sulphate sodium is an established model to study the effects of inflammation on the colon mucosa and immunity, resembling the ulcerative colitis alterations [70,71]. Carrageenan is also used as a colitis inducer [72,73]. Data are summarized in Table 5.

Dextran sulphate sodium (DSS) at the concentration 5% and 1% (*w*/*v*) in drinking water was given for 3 and 14 days respectively to 6–7 weeks old female GF and conventionalized (CVz) (four weeks old GF mice fed with faeces of SPF BALB/c Cr Slc mice) IQI/Jic mice. GF mice treated with 5% DSS, died after three day, had no sign of colitis in the large intestine; however moderate colitis developed in the cecum and proximal colon of CVz mice. GF mice on 14 day after 1% DSS developed severe colitis in the distal colon which was similar to CVz mice after seven days of 5% DSS treatment. In contrast to GF mice, only mild basal crypt loss was observed in the distal colon of CVz mice given 1% DSS [74]. The difference observed between GF and CVz mice were not imputable to DSS degradation by the intestinal microflora in CVz, as DSS is resistant to bacterial degradation [75]. However, Hartley strain male GF guinea pigs did not develop colitis when carrageenan 5% (*w*/*v*) was given in drinking water, contrary to their conventional counterparts treated similarly. This different response further pointed out to the role of intestinal microbiota in the development of colitis [72,76]. An example is the DSS-induced acute colitis which was seen more severe in CV BALB/c mice compared to GF [77]. Using both immunodeficient (Severe Combined Immunodeficiency, SCID) and immunocompetent Balb/c mice, the induction of DSS colitis did not produce changes in the colon mucosa of GF SCID mice, and milder effects in the immunocompetent GF compared to the CV. This underlines the role that active immunity and microbiota relationships have in the development of pathologic conditions in the bowel. The importance of the microbiota composition, independently from the local immunity, has also been demonstrated in immunodeficient mice where some bacterial strains resulted protective against DSS-induced colitis [78]. Monocolonization with E. coli O6K13 or with E. coli strain Nissle 1917 of GF immunocompetent and SCID mice showed how different bacterial strains could differently affect the colitis development both directly and in association with a competent immunity [79]. Once again, the importance of the local microbiome is highlighted in different studies using the same model, even though experimental results sometimes contrasting.

Dextran sulphate sodium in association with AOM is used also for evaluating the contribution of inflammation to the promotion of carcinogenesis [80,81]. SPF C57BL/6 J mice and GF C56BL/6 J mice were injected with 10 mg/kg AOM i.p. for five days. From 6 th day until 10 th day 1% or 1.5% DSS was given in the drinking water (DSS percentage depending on the lot). After that, mice were given untreated drinking water for 16 days. DSS given for 5 days followed by 16 days of untreated drinking water was repeated for two times. Mice were sacrificed 21 days after the last cycle of DSS. AOM/DSS treatment resulted in injured intestinal epithelia and increased intestinal permeability, which led to bacterial translocation into the mucosa. Higher doses of DSS (2.5% or 2%) together with AOM in GF mice showed 100% mortality, and i complete loss of crypts in a significant portion of distal colon was observed in moribund GF mice. However, with lower concentrations of DSS, 100% survival of GF mice was seen. Moreover, in GF mice were observed larger and more numerous adenomatous tumors than in conventional SPF mice. These results suggest that the gut microbiota can even play a protective role against colitis development [82]. The absence of bacteria in GF animals make them more prone to DSS-induced colitis [83]. A potential protection by bacteria came using mono-colonization of GF animals with *Bacteroides fragilis*, with a reduction of the DSS effectiveness in inducing colitis [84]. Differently, the association of AOM and DSS in wild-type mice, or knocked out for molecules involved in the inflammatory network (Myd88, IL10), showed a prevalence of cancer development in the bacterial-associated mice versus the GF group [85]. The type of microbial components may differently modulate the DSS effects as well as the used experimental model.

## 4. Discussion

The evaluation of tumor incidence in experimental animals, prevalently done in the rat and in the mouse, has revealed some interesting insight when comparing GF and CV animals. The intestinal microflora presented a bivalent effect on the tumor development. For example, the spontaneous intestinal polyposis found in GF Balb/c mice suggests that the microflora may have a suppressive activity on its development, since the lower incidence in the CV animals [34]. However, the intestinal microflora enhanced liver tumorigenesis in CV C3H/He male mice [35] and enhances the carcinogenic potential of various chemical inducers of colon cancer experimental models. Therefore, the effect of the microflora can be different depending on the organ undergoing tumorigenesis, in this case the liver and the small intestine. This also indicates that alterations that can affect the composition of the microbiome can differently act on the colon mucosa [23,43]. Effectively, reviewing the literature, different and sometimes contradictory opinions emerged as a result of the increased number of studies and experimental protocols. The expansion of this field has been allowed by the improved rearing of GF mice and rats, with multiplication of the GF facilities around the world. It has also forwarded the knowledge on the commensal microflora composition and its interconnection with the carcinogenesis processes, especially of the colon [13,17,18]. Spontaneous tumors appear with aging and, in GF animals, with higher frequency when they are over one-year-old. They involve predominantly hormonally regulated tissues (breast and even prostate) and immune tissues (leukemia, thymoma). Other tumors can differently occur (e.g., lung), and the gut resulted involved at the small bowel level developing multiple polyposis especially in mice [28,29,30]. The similarity with tumors occurring in human old population or in immune-suppressed patients suggests that in these cases the contribution of the microbiome is basically less relevant than hormonal and immunological changes occurring with aging and immune impairment. Nevertheless, dysbiosis is observed to occur more frequently with the aging and can represent an additionally harmful condition increasing the constitutive risk of degenerative events [86]. Anyway, the “good” and the “bad” of bacteria in cancer development start to be better described due to the discovery of unexpected anti-cancer properties as opposed to the dysbiosis sustaining the carcinogenic processes [87].

Different carcinogens were used to develop various tumor models in GF aniamls. The effects on GF animals resulted variable depending on the organs induced to carcinogenesis and the type of used carcinogen. A bulk of data indicates a lower susceptibility of GF animals than the CV counterparts to develop tumors when DMH or AOM are used, due to the activation of the carcinogen in relation to the microbiome and its composition. More variability was observed with carcinogens like bracken. In this last case, the lower incidence in CV animals suggested a positive role of the microflora by its metabolizing capacity and consequent reduction of the carcinogen’s effect. The induction of colorectal cancer, especially if associated to DSS-induced colitis, has sometimes produced contradictory results [82,85]. Possible reasons may include the variability associated with the experimental protocols (dosages of DSS, timing of administration, AOM dosage, and schedule of administration), as well as withthe choice of the animal strainand its microbiome. Genetic susceptibility is in fact another important factor to be considered in the choice of the animal strain [28], affecting tumor incidence even applying the same protocol of carcinogenesis. The age of induction and the immune system maturation can also affect the induced processes′ outcome, both inflammatory and carcinogenetic. Also, the type and evolution of inflammation, a crucial factor for cancer establishment, can differently sustain the carcinogenesis progression and further responsiveness of the immune system in the GF animals [26,88,89]. The type of diet offers a further element of variability to be considered in the planning and evaluation of the experiments especially in presence of specific requirements. In this regard, particularly relevant are the GF animals, which depend on supplements of vitamins and nutrients wich CV animals receive from the intestinal microflora. Additionally, the level of LPS released from the bacteria in the sterilized food, if not adequately controlled, can affect the GF immunological maturation of the mucosa-associated lymphatic tissue (MALT) and consequently the reactivity of the mucosal immunity, influencing their local anti-cancer responses [88].

Different metabolism of food components (e.g., fats), bile release, and biliary acid re-circulation can modify the response to specific carcinogens by increasing or reducing their effectiveness depending on their elaboration [90]. Similarly, mucosal enzymes result more expressed in GF animals because of the absence of the microflora which downregulates their secretion [66,67].

## 5. Conclusions

The studies on the spontaneous development of tumors in GF animals bringa new insight in the research on the role of microbiome and its relationships with the development of CRC. They result particularly useful in studying how the microbiome, according to its composition, can modulate different carcinogenetic processes, both environmental dependent and physiopatologically related(e.g., aging, inflammation) The data and observations about the induced carcinogenesis coming from the early literature should stimulate the review ofearly hypotheses and results with the support of the presently upgraded technical and methodological approaches. The GF animals represent a very effective tool to better understand both the biological mechanisms regulating the microbiome-host interactions and to dissect the bacterial strains that can directly or indirectly protect against the tumor or viceversa actively promote it. Additionally, they represent an excellent model to study the preventive potential of pre- and probiotics as instrument of cancer intervention.

## Figures and Tables

**Table 1:** Spontaneous tumors in conventional experimental rats and mice.

CV Spontaneous	Incidence %	Main Types of Tumors	Strain	Gender	n. of Animals	Time of Observation	Year	Citation
Rats	45%	endocrine	Sprague-Dawley	♂	179	up to 18 months	1973	[27]
		mammary gland endocrine		♀	181			
Mice	26%	lung	Swiss	♂	101	up to 18 months	1973	[27]
		lung		♀	153			
	36%	mammary gland	C3Hf/Pi	♀	392	up to 24 months	1967	[31]
	37%	small bowel—polyposis	BALB/c	♀	41	12 months	1984	[34]
	51%			♂	51			
	82%	liver	C3H/He	♂	17	11 months (48 weeks)	1979	[35]

Abbreviations—CV: conventional; Balb: Bagg albino mouse strain; C3H: mouse strain and its variants; F344: Fisher 344 rat strain.

**Table 2:** Spontaneous tumors in germ-free (GF) experimental rats and mice.

GF Spontaneous	Incidence %	Main Types of Tumors	Strain	Gender	n. of Animals	Time of Observation	Year	Citation
Rats	25/? *	mamary gland, thymus	wistar	♀, ♂	359	up to 38 months	1963	[28]
	0 **	-	fisher					
	0 **	-	sprague-dawley					
	0%	-	wistar	♀, ♂	33	older than 24 months	1970	[29]
	13%	prostate	wistar	♂	31	20–40 months	1973	30
	19.6% ♀	mamary gland	F344	♀	78♂	from 7 months to >3 years	1976	[12]
	11.5% ♂			♂				
	36% ♀	leukaemia		♀	102♀			
	25.6% ♂			♂				
Mice	30%	mamary gland	C3H/Pi	♀	66	up to 24 months	1967	[31]
	21%	lung	BALB/cPi	♀, ♂	67 (15♂, 52♀)	median age 21 months	1971	[32]
	3%	mamary gland						
	4.5%	lympho-sarcoma						
	3%	reticulum cell sarcoma						
	3%	skin carcinoma						
	3%	adrenal cortical carcinoma						
	1.5%	ovaries		♀				
	14/? *	leukaemia	AK	ns	ns	mean age 8,7 months	1965	[33]
	68%	small bowell—polyposis	BALB/c	♀	40	12 months	1984	[34]
	89%			♂	36			
	39%	liver	C3H/He	♂	49	11 months (48 weeks)	1979	[35]

(*) unknown number of total Wistar rats, here only the absolute number of tumor bearing animals; (**) all animals negative for tumor in the observed strain.

**Table 3:** Tumor occurrence in GF rats and mice induced with chemical carcinogens.

Induced Tumors	Chemical	Method of Administation	Incidence GF %	Incidence CV %	Main Types of Tumors	Strain	Gender	n. of Animals	Age of Animals at the Start of Experiment	Year	Citation
Rats	DMBA + 15% fat emulsion	intra peritoneal	56%	67%	mammary gland, leukaemia	Sprague-Dawley	♂	18 GF (−2†)	1 month	1967	[36]
								11 (−5†) CV			
			100%		myeloid leukaemia	Fisher		18 GF (−16†)			
	DMBA in sesame oil	stomach tube	100%	89%	breast	Sprague-Dawley	♀	11 (−2†) GF	2 months	1963	[37]
								29 (−11†)CV			
			20%	0%		Fisher	♀	8 GF			
								12 CV			
			all died because of cytotoxicity	0%		Wistar	♀	15 (−15†) GF			
								9 CV			
	MCA in olive oil	subcutaneous	79%	82%	fibrosarcoma	Wistar	♀, ♂	14 GF, 11 CV	various	1964	[38]
			96%	87%		Fisher	♀, ♂	27 GF, 39 CV			
Mice	MCA in olive oil	subcutaneous	100%	100%	fibrosarcoma	Swiss-Webster	♀, ♂	13 GF, 14 CV	various	1964	[38]
			80%	72%		C3H	♀, ♂	71 GF, 18 CV			
			57%			ICR	♀, ♂	33 GF			
			76%			CFW	♀, ♂	17 GF			
	20-MCA in olive oil	orally	94%		leukaemia	RFX	♀, ♂	48 GF	5–6 weeks	1971	[39]
			27%		lung			49 GF			
	20-MCA + croton oil	Orally—topically (abdomen skin)	97%		leukaemia			68 GF			
			44%		lung			62 GF			
	MCA in olive oil	subcutaneous	100%	89%	lung	Swiss-Webster	♀, ♂	61 GF, 54 CV	newborn	1963	[40]
	Urethane	intra peritoneal	56%	85%	lung	BALB/c	♀	17 GF, 20 CV	6–9 weeks	1970	[41]
			0%	25%		C3H/HeN	♀	4 GF, 20 CV			
			0%	27%		C57Bl/6N	♀	10 GF, 22 CV			
			0%	14%		C57Bl/6N	♂	3 GF, 22 CV			
	mineral oil	intra peritoneal	6%	70%	plasma cell	BALB/c AnN	♀	33 GF, 40 CV	1–2 months	1969	[42]
			6%	5%	pulmonary adenoma						
			48%	10%	pleomorfic reticulum sarcoma						

Acronym of chemicals. DMBA: 7, 12-dimethylbenz(a)-anthracene; MCA: Methylcholanthrene.

**Table 4:** Colorectal cancer induction by carcinogens in GF rats and mice.

Induced Tumors	Dietary Component/Carcinogen	Method of Administation	Incidence GF %	Incidence CV %	Number of CRC Tumors/Animal	Type of Tumor—Specified	Strain	Gender	n. of Animals	Age (Weight) of Animals at the Start of Experiment	Year	Citation
Rats	MNNG (increasing from 1 mg to 3 mg/rat and week, 20 weeks)	intra-rectal	100%	100%	5.8 ± 0.58 GF		Fisher	♀	24 GF	50 days	1975	[49]
					3.00 ± 0.31 CV				23 CV			
	MNNG (100 μg/mL)	in drinking water ad libitum	17%	91%	ns		Wistar	♂	30 GF, 33 CV	80–95 g	1979	[50]
	MNNG (16 mg/rat total dose)	intra-rectal	89%		2.7 ± 0.39		Fisher 344	♀	16 GF	7 weeks	1976	[14]
	MNNG (16 mg/rat) + DC (3 g/rat total dose)		82%		4.2 ± 0.5				22 GF			
	DMH (20 weeks)—sacr. after last dose	subcutaneous	0%	17%	ns		CD Fisher	♀	12 GF, 12 CV	7 weeks	1974	[48]
	DMH (20 weeks)—sacr. 20 weeks after last dose (40 weeks total)		11%	25%					18 GF, 24 CV			
	DMH (20 mg/kg/week; 20 weeks)	intra-rectal	43%	86%	1.0 ± 0.1 GF		Fisher	♀	28 GF	7 weeks	1976	[62]
					2.1 ± 0.2 CV				28 CV			
	AOM (10 mg/kg/week; 20 weeks)	subcutaneous	100%	60%	7.4 ± 0.8 GF				20 GF			
					1.1 ± 0.2 CV				28 CV			
	Bracken	mixed with diet	90%	94%		all CRC tumors	Wistar	♀	10 GF, 16 CV	80–90 g	1981	[68]
			70%	50%		adenoma						
			0%	63%		adenocarcinoma						
			90%	31%		sarcoma						
	AOM (9 mg/kg), 1x week, 5 weeks + bile (3 mL—3x week, 3 weeks)	subcutaneous + intracecal (surgicaly created cecal hernia)	50%	77.7%	1 GF		Wistar-AVN	♂	10 GF	150 g	2008	[28]
					1.85 CV				10 CV			

Acronyms of chemicals. MNNG: Methylnitronitrosoguanidine; DC: deoxycholate; DMH: 1,2 dimethylhydrazine; AOM: azoxymethane.

**Table 5:** Examples of carcinogenesis with AOM and induced colitis in GF animals.

Species	Chemical	Method of Administation	Incidence GF %	Incidence CV/CVz %	Medical Conditions	Strain	Gender	n. of Animals	Age (Weight) of Animals at the Start of Experiment	Year	Citation
Mice	DSS 5% and 1%	Orally—drinking water	84%	10%	colitis	IQI/Jic	♀	19 (−6†) GF	6–7 weeks	2001	[74]
								10 CVz			
	DSS 2.5%	orally—drinking water	53%	44%	acute and chronic colitis	BALB/c		15 (−5†) GF	6–7 weeks	2001	[77]
								18 (−4†) CV			
			75%	86%		SCID		16 (−8†) GF			
								14 (−6†) CV			
	AOM (10 mg/kg), DSS 1–2.5%	orally—drinking water	100% *	0% *	colitis	C57BL/6		GF	6–12 weeks	2013	[82]
								CV			
Guinea pigs	Carrageenan 5%	orally—drinking water	0%	100%	Cecal ulceration	Hartley strain guinea pigs	♂	6 GF	250–300 g	1981	[72]
								142 CV			

**Acronyms:**.DSS: dextran sodium sulphate; AOM: azoxymethane; SCID: severe combined immundeficiency. * mortality after DSS >2%

## Data Availability

All reported data resulted from publication in PubMed, WoS, Scopus and all used articles are reported in the References.
